# Urinary Deposits, Their Diagnosis, Pathology and Therapeutical Indications

**Published:** 1846-01

**Authors:** 


### REVIEWS.

---

ART. V.—*Urinary Deposits, their Diagnosis, Pathology, and Therapeutical Indications.* By *Golding Bird, A.M., M.D.*, Assistant Physician to, and Lecturer on Materia Medica at, Guy's Hospital; Licentiate of the Royal College of Physicians; late President of the Westminster Medical Society; Fellow of the Linnean Society of London; Corresponding member of the Philosophical Institute of Basle, of the Philosophical Society of St. Andrews, of the Medical Society of Hamburgh, &c., &c., &c. Philadelphia: Lea & Blanchard. 1845. 8vo. pp. 227.

The author of this volume is at once a chemist skilled in analysis, and a practitioner who has for years carefully noted diseases at the bedside. It is therefore manifest, that he is qualified in an uncommon degree to discuss the subject of urinary deposits, in which the phenomena belong as much to chemistry as to pathology. Such are the laborers from whom science is likely to derive the most valuable results, as to all the pathological conditions which involve chemical reactions. The mere chemist is not competent to the task of unfolding them; and the pathologist without the tests and reagents of the laboratory is unable to account for the series of changes. The union of the two, as it is found in Dr. Bird, is indispensable to a successful prosecution of such researches. Chemistry places in his hands instruments for detecting morbid products, and for estimating the departure of the various fluids of the body from a state of health, and clinical observation answers as a check upon the speculations of the laboratory. But to the work before us, of which it is our purpose to attempt an abstract in this and a succeeding number of our Journal.

The author observes, in his preface: "In coming in contact with pupils in the course of my duties as a teacher of my profession, and in mixing with medical men, in practice, I have often found them in want of some work which would enable them readily to discover the nature of a deposit in the urine, and succinctly point out its pathological and therapeucal indications." It is as a manual for the practitioner in urinary affections that he presents his work to the profession, and in that character it has the highest claims to our attention. Its matter is condensed, and so arranged that ready reference may be made to any topic.

The following observations relative to the superficial examination of the urine in the sick room will be found of great value by the practitioner:

"A piece of litmus paper should be immersed in the urine, which, if acid, will change the blue color of the paper to red. Should no change occur, a piece of reddened litmus paper must be dipped in, and if the secretion be alcaline, its blue color will be restored; but if no change occur, the urine is neutral.

"Some of the urine should then be gently heated in a polished metallic spoon over a candle, or what is preferable, in a test-tube over a spirit-lamp, and if a white deposit occurs, albumen or earthy phosphates are present; the former, if a drop of nitric acid does not redissolve the deposit, the latter if it does.

"If the urine be very highly colored, and undergoes no change by boiling, the coloring matters of bile, blood, or purpurine are present. To determine which, pour a thin layer of urine on the back of a white plate, and allow a few drops of nitric acid to fall in the centre; an immediate and rapidly ending play of colors, from green to red, will occur if bile, but no such change if purpurine alone exists. Should the highly colored urine alter in color or transparency by heat, the presence of blood must be suspected.

"If the addition of nitric acid to deep red urine, unaffected by heat, produces a brown deposit, an excess of uric acid exists. If the urine be pale, immerse the gravimeter, and if the specific gravity be below 1·012, an excess of water exists in the urine, but if above 1.025, the presence of sugar, or excess of urea is indicated. To determine which, place a few drops in a watch-glass, add an equal quantity of nitric acid, and allow the glass to float on some cold water; crystalization of nitrate of urea will occur in two or three minutes, if the latter exists in excess. Should this change not occur, the urine must be examined specially for sugar, which, it must be remembered, may exist in small quantities, without raising the specific gravity of the fluid.

"Should the urine be alcaline, add a drop of nitric acid; if a white deposit occurs, albumen is present; if brisk effervescence follows the addition of the acid, the urea has been converted into carbonate of ammonia.

"*Urine depositing a visible sediment.*

"If the deposit is flocculent, easily diffused on agitation, and scanty, not disappearing on the addition of nitric acid, it is chiefly made up of healthy mucus, epithelium, or in women, an admixture of leucorrhœal discharge.

"If the deposit is ropy and apparently viscid, add a drop of nitric acid; if it wholly or partially dissolves, it is composed of phosphates, if but slightly affected, of mucus. If the deposit falls like a creamy layer to the bottom of the vessel, the supernatent urine being coagulable by heat, it consists of pus.

"If the deposit is white, it consists of urate of ammonia, phosphates, or cystine; the first disappears on heating the urine, the second on the addition of a drop of diluted nitric acid, whilst the third dissolves in ammonia, and the urine generally evolves an odor of sweet-briar.

"If the deposit be colored, it consists of red particles of blood, uric acid, or urate of ammonia, stained with purpurine. If the first, the urine becomes opake by heat; if the second the deposit is in visible crystals; if the third, the deposit is amorphous, and dissolves on heating the fluid.

"Oxalate of lime is often present diffused through urine, without forming a visible deposit; if this be suspected, a drop of the urine examined microscopically will detect the characteristic crystals.

"Much of the little time required for the investigation thus sketched out, may be saved by remembering the following facts:

"If the deposit be white, and the urine acid, it in the great majority of cases consists of urate of ammonia; but should it not disappear by heat, it is phosphatic.

"If a deposit be of any color inclining to yellow, drab, pink, or red, it is almost sure to be urate of ammonia, unless visibly crystalline, in which case it consists of uric acid.

"The only apparatus and re-agents required for these investigations at the bedside are—

"A gravimeter, made small enough to float in an ounce of fluid.

"Red and blue litmus paper.

"A test-tube and watch-glass.

"Nitric acid."

The urine is affected by a variety of circumstances, and varies with the food and drink, and with the condition of the body. Dr. Bird recognizes three varieties of this secretion.

"Firstly, that passed some little time after drinking freely of fluids, generally pale, and of low specific gravity, (1.003—1.009) *urina potus.* Secondly, that secreted after the digestion of a full meal, varying much in physical characters and of considerable destiny: (1.020—1.028 or even 1.030,) *urina chyli vel cibi.* Thirdly, that secreted from the blood independently of the immediate stimulus of food and drink, as that passed after a night's rest, *urina sanguinis;* this is usually of average density, (1.015—1.025,) and presents in perfection the essential characters of urine."

To ascertain the specific gravity of the urine—

"If a gravimeter be not at hand, any small stoppered phial may be substituted. For this purpose, counterpoise the empty bottle and stopper in a tolerably good balance, with shot or sand. Then fill it with distilled water, insert the stopper, and carefully ascertain the weight of the water it contains. Empty the bottle, fill it with urine, and again weigh it; the specific gravity of the fluid will be readily found by merely dividing the weight of the urine by that of the water."

The chemical composition of the urine in health is as follows:

I. ORGANIC PRODUCTS.

| | |
|---|---|
| 1st. Ingredients characteristic of the secretion, produced by the destructive assimilation of tissues, and separated from the blood by the kidneys. | Urea, uric acid, coloring and odorous principles. |
| 2d. Ingredients developed principally from the food during the process of assimilation. | Hippuric acid, lactic acid? accidental constituents. |

II. SALINE PRODUCTS.

| | |
|---|---|
| 3d. Saline combinations separated from the blood, and chiefly derived from the food. | Phosphates, Chloride, of sodium. |
| 4th. Saline combinations chiefly generated during the process of destructive assimilation. | Sulphates. |

III. INGREDIENTS DERIVED FROM THE URINARY PASSAGE.

5th. Mucus of the bladder.
6th. Debris of epithelium.

"Of these, the first class of ingredients can alone be considered as really essential to the urine, and characteristic of it as a secretion, the kidneys being the only organs which normally secrete these elements from the blood. The saline ingredients of the second class are met with in most secretions of the body, with the exception of the sulphates, which are rarely found except in the urine. The third class of elements is met with in all fluids passing over mucous surfaces."

The urea, which is the characteristic ingredient of urine, bears a striking proportion to the quantity of azote taken in the food, as appears from the following table, copied by our author from Dr. Lehmann, of Leipsic:

| Diet. | Animal. | Vegetable. | Mixed. | Non-nitrogenised. |
|---|---|---|---|---|
| Urea in the urine, of 24 hours. | 819.2 | 346.5 | 500.6 | 237.1 |

Among the other constituents of urine is sulphuric acid, which Dr. Bird maintains is found in too large a quantity to be explained by its presence in the food, and which, therefore, he refers to the oxydation of sulphur, one of the elements of the protein compounds.

The urine, which in health, and while its constituents maintain their proper relation to each other, is clear and of a pale amber color, becomes turbid when any of its ingredients exist in a comparative or real excess. All the deposits found in it may be divided according to our author into the four following classes:

"*Class* 1.—Deposits composed essentially of ingredients formed directly or indirectly from the metamorphosis of tissues, or from the organic elements of food.

Uric acid and urates.
Uric oxide.
Oxalate of lime.
Cystine.

"*Class* 2.—Deposits composed of ingredients of inorganic origin; including—

Phosphate of lime.
Ammonio-phosphate of magnesia.
Carbonate of lime.
Silicic Acid.

"*Class* 3.—Highly colored deposits (black or blue) of doubtful origin,

Cyanourine.
Melanourine.
Indigo.
Prussian blue.

"*Class* 4.—Deposits consisting of non-crystalline organic products; including—

A. *Organized.*
Blood.
Pus.
Mucus.
Organic globules.
Epithelium.

B. *Non-organized.*
Milk.
Fatty matter.

c. *Possessing independent vitality.*
Spermatozoa.
Torulæ.
Vibriones."

The first deposit described is the *uric acid*, the diagnosis of which is thus given:

"When heated in the urine, the uric acid deposit does not dissolve; the crystals merely become opaque. It generally becomes more distinct from the solution of urate of ammonia, which is frequently mixed with it, and sometimes completely conceals it from view. Hence the best mode of discovering this deposit, is to warm urine, turbid from excess of urate of ammonia, in a watch-glass; the acid becomes visible in the centre of the glass, as soon as the urate dissolves. Heated with liquor potassæ, the uric acid deposit dissolves, from the formation of an urate of potass of sparing solubility. Hydrochloric and acetic acids are without any action, but the nitric readily dissolves it, and by careful evaporation a residue of a beautiful pink color becoming of a rich purple, on being laid over the vapor of ammonia, is left. This colored residue is the murexid of Liebig, the purpurate of ammonia of Dr. Prout. Exposed to heat in a platinum spoon, the uric acid deposits burn, evolving an odor of bitter almonds; and finally leave a small quantity of a white ash, which generally contains phosphate of soda or lime.

"*Characters of urine depositing uric acid.*—When urine contains an excess of this acid, it generally lets fall crystals on cooling, uric acid being very seldom deposited before emission. It usually possesses a deeper amber tint than natural, sometimes being of a reddish brown color. Very high colored urine, however, seldom deposits uric acid until after the addition of a stronger acid. Urine never lets fall spontaneously all its uric acid as a deposit, for after being filtered from a sediment of this substance, the addition of a drop of nitric acid generally causes the deposition of an abundance of crystals of uric acid in a few hours.

"Urine depositing uric acid always reddens litmus paper, and often contains an excess of urea, so as to crystalize slowly when mixed with nitric acid in a watch-glass. Its specific gravity is generally rather above 1.020. An exception to the above character is presented by the pale urine of infants at the breast, among whom deposits of uric acid are common. These often appear as a yellow crystalline sand, whilst the supernatant urine is frequently of low specific gravity, often 1.006, as pale as water, and nearly destitute of urea."

Urate of ammonia, another of the urinary deposits, is recognized by the following characters:

"These deposits vary in color from absolute whiteness to a pale fawn color, which is the most frequent tint, brick-red, pink, or purple. All these various colored deposits present certain characters in common; they never appear in the urine until after it has cooled, and disappear with the greatest readiness on the application of heat. The purple deposits require rather a higher temperature for solution than the other. The addition of liquor ammoniæ, or liquor potassæ, immediately dissolves deposits of urate of ammonia. Their chemical constitution is shown in a very interesting manner by examining a drop of the turbid urine with the microscope between two plates of glass; an amorphous powder will be alone visible, unless uric acid be present; then add a drop of hydrochloric acid, the turbidity will disappear, and in a short time crystals of uric acid will be seen growing in the fluid, the ammonia having deserted this substance to unite with the acid which had been added.

"*Characters of urine depositing urate of ammonia.*—The following modifications are most important.

"1st. A pale urine of low specific gravity (1.012), becoming opaque on cooling from the deposition of nearly white urate of ammonia, which, instead of readily falling, forms rope-like masses in the fluid, and presents on a superficial view so much the appearance of muco-pus as to be mistaken for it. Its disappearance on the application of heat at once will discover the error.

"2d. A pale amber colored urine of moderate density (1.018), which on cooling lets fall a copious fawn colored deposit, resembling bath-brick grated into the urine, disappearing with the utmost readiness on applying a gentle heat. This deposit is of frequent occurrence, is often very transient, and is so constantly an attendant on the slightest interference with the cutaneous transpiration, that a 'cold' is popularly diagnosticated whenever this state of things exists.

"3d. Whenever febrile excitement prevails, the urine becomes concentrated, rises in density (1.025,) and deposits on cooling a reddish brown sediment, constituting the well-known lateritious, or brick dust sediment. This variety of urine generally becomes turbid on the addition of a drop of nitric acid, not from the coagulation of albumen, as has been frequently erroneously supposed, but from the precipitation of uric acid. This is always in minute microscopic crystals, notwithstanding the amorphous appearance it presents to the naked eye.

"4th. In well-marked affections of the portal circulation, especially when connected with organic disease of the liver or spleen, or when suppuration, particularly of a strumous character, is going on in the body, the urine is generally found to possess in many instances a deep purple or copper color, often verging on crimson, so as to have led to the idea of blood being present. These deep tints appear to me to depend upon the presence of an excess of purpurine.—Whenever a deposit of urate of ammonia occurs in such urine, either spontaneously or by immersing it in a freezing mixture, it combines with the pink pigment forming a kind of lake, and which is often so abundant as not to entirely disappear by heat, until the urine is diluted by the addition of water. These deposits do not exhibit their delicate tints until after being collected in a filter; they readily give up their coloring matter to alcohol, which leaves their urate of ammonia nearly unchanged."

[TO BE CONTINUED.]

